# Human rights and the WHO FCTC Conference of the Parties

**DOI:** 10.18332/tid/130786

**Published:** 2020-12-08

**Authors:** Kelsey Romeo-Stuppy, Stephen Marks, Pablo Analuisa, Flavia Senkubuge, Laurent Huber

**Affiliations:** 1 Action on Smoking and Health, Washington, United States; 2 François-Xavier Bagnoud Center for Health and Human Rights, Harvard T.H. Chan School of Public Health, Boston, United States; 3 Ministerio de Salud Pública, Quito, Ecuador; 4 School of Health Systems and Public Health, Faculty of Health Sciences, University of Pretoria, Pretoria, South Africa

**Keywords:** human rights, tobacco, WHO Conference of the Parties

## Abstract

‘In the absence of the COVID-19 pandemic, many people in tobacco control worldwide would have been at the Hague, Netherlands, from 9–14 November for the 9th Conference of the Parties (COP9) of the WHO Framework Convention on Tobacco Control (FCTC), advocating for even stronger policies against the tobacco epidemic. The COP has been postponed to 2021, but the pandemic did not stop the global civil society from ‘virtually’ gathering to talk about the FCTC, where it is and where it is going.’

The COVID-19 pandemic has killed 1.33 million people worldwide, which is both a public health disaster and a massive failure to protect the right to health. COVID-19 reveals another human rights dimension, that of its disproportionate impact on the most vulnerable members of society, thus exacerbating racial and social injustice. Both of these issues, and many more, are intrinsically connected to tobacco control through human rights.

For example, in the United States, because of targeted marketing from the tobacco industry, African Americans are significantly more likely to smoke menthol cigarettes – nearly 9 out of 10 African American adults who smoke, smoke menthol^1^. The African American population is also significantly more likely to catch COVID-19 (2.5 × higher) and to die of it (2.1 × higher)^2^. Tobacco use, COVID, social justice, and human rights are all intricately tied together, and therefore the response must be multi-faceted and holistic, as well as at the local, national and global levels.

A human rights approach to tobacco control is much more than an academic or theoretical concept; it was envisioned early on in the negotiations of the FCTC and in the final text, which makes explicit reference to human rights treaties^3^. Building on that affirmation, actors in the public health community, international and national agencies, and civil society, are beginning to put the human rights approach into practice.

Action on Smoking and Health, the African American Tobacco Control Leadership Council and the American Medical Association recently filed a lawsuit against the United States Food and Drug Administration for its inaction on menthol cigarettes^4^. In addition to utilizing the court system, advocates are encouraging the Committee on the Elimination of Racial Discrimination (CERD), an international human rights treaty body, to take action based on non-discrimination and the right to health^5^. This case is part of a broader effort at the local and international levels to combine tobacco control and human rights efforts to tackle the sale of menthol cigarettes in the US.

The FCTC is not generally considered to be a human rights treaty, but instead is viewed as a public health treaty as it was negotiated under the auspices of the World Health Organization. Making a more formal linkage between the FCTC and human rights objectives would highlight the mutually reinforcing nature of tobacco control and the human rights agenda and support tobacco control advocacy around the world. Recognition of the value of this linkage has motivated several governments and civil society groups to join forces towards a human rights decision at COP9. Such a decision is likely to request the Convention Secretariat to:

- Improve coordination with the Office of the High Commissioner for Human Rights;
- Cooperate with human rights mechanisms, including treaty bodies, special procedures and the Universal Periodic Review;
- Collaborate with the Intergovernmental Working Group negotiating a treaty on TNCs and human rights.

Such a decision could also urge FCTC parties to implement Target 3a of the SDGs^6^ from a human rights perspective, and include tobacco control in their reporting under human rights treaties.

A human rights decision at COP9 could bolster tobacco control efforts in litigation, implementing FCTC best practices and in many other ways. A human rights decision could help make linkages across issue areas to garner the support we need to end the tobacco epidemic for good. The first step is to ensure that we get a strong human rights decision at COP9.
